# Foreign Language Learning as Cognitive Training to Prevent Old Age Disorders? Protocol of a Randomized Controlled Trial of Language Training vs. Musical Training and Social Interaction in Elderly With Subjective Cognitive Decline

**DOI:** 10.3389/fnagi.2021.550180

**Published:** 2021-04-27

**Authors:** Saskia E. Nijmeijer, Marie-José van Tol, André Aleman, Merel Keijzer

**Affiliations:** ^1^Cognitive Neuroscience Center, Department of Biomedical Sciences of Cells and Systems, University Medical Center Groningen, University of Groningen, Groningen, Netherlands; ^2^English Linguistics and English as a Second Language, Bilingualism and Aging Lab, University of Groningen, Groningen, Netherlands

**Keywords:** foreign language learning, bilingualism, cognitive flexibility, cognitive reserve, aging, late-life depression, subjective cognitive decline, functional neuroimaging

## Abstract

**Introduction**: With aging comes a reduction of cognitive flexibility, which has been related to the development of late-life depression and progression of general cognitive decline. Several factors have been linked to attenuating such decline in cognitive flexibility, such as education, physical exercise and stimulating leisure activities. Speaking two or more languages has recently received abundant attention as another factor that may build up cognitive reserve, thereby limiting the functional implications of compromised cognition that accompany old age. With the number of older adults reaching record levels, it is important to attenuate the development of old-age disorders. Learning to speak a foreign language might offer a powerful tool in promoting healthy aging, but up to date effect studies are sparse. Here, the protocol that forms the foundation of the current study is presented. The present study aims to: (1) examine the effects of a foreign language training on cognitive flexibility and its neural underpinnings, and on mental health; and (2) assess the unique role of foreign language training vs. other cognitive or social programs.

**Method**: One-hundred and ninety-eight Dutch elderly participants reporting subjective cognitive decline are included and randomized to either a language intervention, a music intervention, or a social control intervention. During 3 to 6 months, the language group learns English, the music group learns to play the guitar and the social group participates in social meetings where art workshops are offered. At baseline, at a 3-month follow-up, and at 6 months after termination of the training program, clinical, cognitive and brain activity measurements (combined EEG and fNIRS methods) are taken to assess cognitive flexibility and mental health.

**Discussion**: This is the first trial addressing combined effects of language learning in elderly on cognition, language proficiency, socio-affective measures, and brain activity in the context of a randomized controlled trial. If successful, this study can provide insights into how foreign language training can contribute to more cognitively and mentally healthy years in older adulthood.

**Clinical Trial Registration**: The trial is registered at the Netherlands Trial Register, July 2, 2018, trial number NL7137. https://www.trialregister.nl/trial/7137.

## Introduction

A large portion of the population in Europe speaks more than one language (European Commission Statistics Explained, 2017). Speaking and knowing multiple languages has social benefits, such as being able to communicate with more people. Moreover, research in the past two decades has indicated that cognitive ramifications may also result from bilingual experiences: Speaking multiple languages may contribute to cognitive reserve. This may in turn limit functional implications of cognitive decline that accompanies later life stages. As a function of cognitive decline, older adults typically show decreased cognitive flexibility (Roldán-Tapia et al., 2017; Wilson et al., 2018), which is further weakened in elderly with depression (Rajtar-Zembaty et al., 2017). Overall, the link between late-life depression and cognitive decline has been well documented (Korczyn and Halperin, 2009; Dias et al., 2020). Because of the prevalence of these disorders and the increase in life expectancy in general, they are major contributors to lower quality of life and increased care costs associated with aging (Ganguli et al., 2002; Lenze et al., 2005; Leibson et al., 2015). Attenuating the development of old-age disorders is therefore a major clinical and societal challenge. Given that stronger cognitive flexibility has been reported in bilingual older adults (Gold et al., 2013), learning a foreign language might offer a powerful tool in combating age-associated disorders.

Speaking multiple languages has been claimed to improve cognitive control. For instance, studies comparing monolinguals and bilinguals found bilingual individuals to outperform their monolingual peers in the realms of inhibition (Bialystok et al., 2004), monitoring (Costa et al., 2009) and switching (Garbin et al., 2010; Prior and MacWhinney, 2010). The improvement in cognitive control resulting from speaking multiple languages might ultimately contribute to cognitive reserve (van den Noort et al., 2019a): the capacity of the brain to cope with brain changes such as those occurring in old age (Stern, 2002). Indeed, speaking multiple languages has been associated with the attenuation of cognitive decline: the onset of neurodegenerative diseases like Alzheimer’s disease has been found to be delayed with an average of 4 years (Craik et al., 2010; Alladi et al., 2013; Woumans et al., 2015; Borsa et al., 2018; but see Mukadam et al., 2017; van den Noort et al., 2019b).

The improvement in cognitive abilities relates to the complexity of using and managing multiple languages in one mind. Using multiple languages is believed to result in cross-language competition, where one language interferes with another (Kroll and Gollan, 2013). This language competition occurs for lifelong multilinguals, but is also attested in early language learning phases: interference between the native language (L1) and second language (L2) sets in rapidly after starting an intensive language course (McLaughlin et al., 2004; Sullivan et al., 2014; Bice and Kroll, 2015). Although the underlying processes are not yet crystallized, the conception is that domain-general, rather than language-specific, cognitive control processes are required to manage and resolve such language competition, ultimately strengthening cognitive control.

The improvement in cognitive control that might result from speaking multiple languages is perhaps best captured in terms of cognitive flexibility (Kroll and Bialystok, 2013). Common models of executive functioning often postulate cognitive flexibility, or “mental set-shifting,” as one of the core executive functions (Miyake et al., 2000). However, the process of cognitive flexibility not only involves shifting, but also encompasses inhibition, monitoring, working memory and attention (Dajani and Uddin, 2015). For cognitive flexibility to be successful, these processes of cognitive control must act coherently.

Whether speaking multiple languages indeed improves cognitive flexibility, and if so, under which conditions this happens, is a topic currently strongly debated. Recent meta-analysis studies are inconclusive about the robustness of what has been dubbed a bilingual advantageand the topic has been fiercely debated in recent years (Lehtonen et al., 2018; van den Noort et al., 2019a). Building on what is recommended in earlier studies, it is worth investigating such language-related effects in a controlled way.

One way of shedding light on the impact of bilingual language experiences on cognition and at the same time controlling individual differences in bilingual experiences as much as possible is through organizing foreign language-training courses for (functionally) monolinguals. Earlier work has shown foreign language courses to result in functional and structural brain changes in areas such as the dorsal medial frontal gyrus (MFG), inferior frontal gyrus (IFG), superior frontal gyrus (SFG) and hippocampus (Mårtensson et al., 2012; Sullivan et al., 2014; Bellander et al., 2016; Bubbico et al., 2019). Especially the MFG and IFG are of interest here as they are key areas in adequate cognitive flexibility (Dajani and Uddin, 2015). Interestingly, foreign language training may incur changes in the brain while effects may go undetected in behavioral paradigms (McLaughlin et al., 2004; Kousaie and Phillips, 2017). However, this line of work has almost exclusively targeted younger adults, whereas improvements in brain areas related to cognitive flexibility might be particularly beneficial for older adults. Language learning studies in elderly—in the so called-third age—and their effects are only scantly available (as discussed in detail below).

Tapping into cognitive flexibility and including aspects of novelty are two important factors for cognitive training interventions in establishing long-lasting domain-general effects (Buitenweg et al., 2012). Learning a new foreign language pertains to both factors. Language learning as cognitive training has been hypothesized to benefit elderly in general, but especially seniors with late-life depression or in early stages of dementia may show an attenuation of age-related cognitive decline (Antoniou et al., 2013). Besides that, foreign language training in third age may well hold a special position in promoting healthy aging as it may also incur socio-affective effects. This combination is important when aiming for a broad implementation in reducing the risk of multiple common old-age disorders—such as late-life depression and dementia—simultaneously.

Although in the past older adults were often deemed to be unsuccessful at novel language learning—due to the alleged critical period in language learning (see Singleton, 2005; Muñoz and Singleton, 2011 for an overview), recent studies have found that even at an advanced age, language learning can be successful (Ware et al., 2017; Kliesch et al., 2018). The few language training studies targeting seniors are, however, inconclusive regarding effects on cognitive or socio-affective factors

(see Pot et al., 2019 for a review). Some studies found improvements in self-esteem and well-being but did not look into cognitive effects (Ware et al., 2017; Kliesch et al., 2018). Others found improvements in attentional switching but did not test socio-affective outcomes (Bak et al., 2016). Moreover, only one study found a small improvement in inhibition and well-being (Pfenninger and Polz, 2018). Others, however, found no improvements in cognitive abilities, such as switching (Ramos et al., 2016) or working memory (Berggren et al., 2020), but also did not include socio-affective measures. At present, there has only been one study that examined cognitive improvements in combination with brain changes, as a function of old age language learning: Bubbico et al. (2019). They observed increased functional connectivity and a related improvement in global cognition, but again no socio-affective measures were included (Bubbico et al., 2019). The mixed findings in earlier work can be explained on methodological grounds: the difference in set-up between these studies is substantial. Training periods varied from 1 week to 8 months and the use of control groups varied between the use of only a passive control group, only an active group or a combination of both. What is currently lacking is a thoroughly designed comprehensive study that investigates language proficiency gains, socio-affective measures, cognitive effects, and brain changes in one design such that it allows for the uniqueness of language learning compared to other socio-cognitive interventions to be examined.

Whether language learning has a unique effect on cognition can be studied by comparing it to learning a skill that is closely related. Music is very similar to language: it is complex, contains hierarchical relations, pitch, and contours. On top of that, music and language are said to rely on involvement of similar neural control networks (Jäncke, 2009). Similarly to language learning, music training in which one learns to play an instrument, has been related to enhanced quality of life, mood state, cognition and working memory (Seinfeld et al., 2013; Janus et al., 2016; D’Souza et al., 2018; Schneider et al., 2019). The crucial difference between a language and music intervention is the interference effect that occurs when learning a new language (Bice and Kroll, 2015). Due to the interference that results from language competition, cognitive flexibility is hypothesized to be trained to a larger extent, but such a potential cognitive improvement can only be assigned to language learning when there is no such effect that emerges from the music condition. Additionally, improved well-being on its own might also modulate cognitive performance (Pot et al., 2018). Since both language as well as musical training have tentatively proven to enhance well-being, self-esteem and social interaction (Seinfeld et al., 2013; Ware et al., 2017; Pfenninger and Polz, 2018), it is important to include a no training social control condition to distinguish the social and cognitive contributions of all intervention conditions.

In the current study, the unique effect of a foreign language training program in elderly at risk for late-life depression and cognitive decline (elderly with subjective cognitive decline; SCD) is evaluated in the context of a randomized controlled trial. The trial includes three arms to compare language learning to other complex skill training (musical training) as well as social interaction. The design allows for within- and between-subject comparisons of cognitive flexibility and its neural underpinnings as well as mental health outcomes.

The first objective is to examine the effects of a foreign language training on cognitive flexibility. This is behaviorally examined using several cognitive tasks. Language learning is hypothesized to contribute to cognitive reserve by promoting cognitive flexibility. Neuroimaging methods are used to complement these behavioral methods in a more explorative nature. They provide additional information on how the changes in cognitive flexibility may occur and therefore on the mechanisms by which language training may lead to cognitive advancements providing a more complete picture. Electroencephalography (EEG) and functional Near-Infrared Spectroscopy (fNIRS) are used as complementary methods to obtain both spatial and temporal data (Shibasaki, 2008). It is expected that the language training will induce changes in neurophysiological correlates of cognitive flexibility. Changes in brain activity as a result of the language training are specifically expected in the dorsolateral (dlPFC) and ventrolateral (vlPFC) prefrontal cortex, measured with fNIRS. The lateral prefrontal cortex is associated with both language production, cognitive flexibility, and adequate regulation of mood states (Antoniou et al., 2013; Dajani and Uddin, 2015; Kim et al., 2019) and has been found to function suboptimally in individuals suffering from subjective cognitive decline (Viviano and Damoiseaux, 2020). Language learning is furthermore hypothesized to change event-related frequency band power, which is indicative of activity-related (de-)synchronization of oscillatory activity (Ward, 2003), measured using EEG. The present study primarily considers the theta frequency band, due to its proven relation with cognitive flexibility (González-Hernández et al., 2002; Doesburg et al., 2013; Yeung et al., 2016). A further sub-aim of this study is to examine effects of language learning on psychological and cognitive health. It is expected that the changes in cognitive flexibility will ultimately lead to improvements in subjective cognitive decline, depressive symptoms, and general well-being.

The second objective is to assess the unique role of foreign language training vs. other cognitive training programs or other social engagement activities in establishing the above-mentioned effects. To this end, cognitive flexibility outcomes to ensue from the language training are compared to a high-level active control group in which participants learn to play a musical instrument (guitar) and to a low-level active social intervention (in the form of creative workshops; for more details see below). Because of the similarities and difference between language and music, any additional boost of foreign language training compared to learning other complex skills can be uniquely assessed. To resolve the interference that learning a new language incurs, cognitive flexibility is expected to improve most in the language intervention. Compared to the social intervention, the music intervention is expected to lead to greater effects on cognitive flexibility and (mental) health outcomes (i.e., language > music > social; Park et al., 2014). However, as described, language, music and social activities have all been related to improved well-being and cognition in seniors. Differences among the groups might therefore be small and not significant.

Given its scope and size as well as design, the current study can shed more light on the effects of foreign language training in seniors. Previous studies on lifelong bilingualism as well as foreign language training effects in younger populations demonstrate the relevance and urgency of conducting such a study, whose potential clinical effects are also examined.

## Materials and Analysis

The protocol that forms the foundation of the current study is presented. This study is funded by the University of Groningen and the University Medical Center Groningen (UMCG) and approved by the medical ethical board of the UMCG (registration number NL65233.042.18). The trial is registered at the Netherlands Trial Register (NTR), protocol number NTR7336. All subjects give written informed consent in accordance with the Declaration of Helsinki.

### Study Design

**Figure 1:**
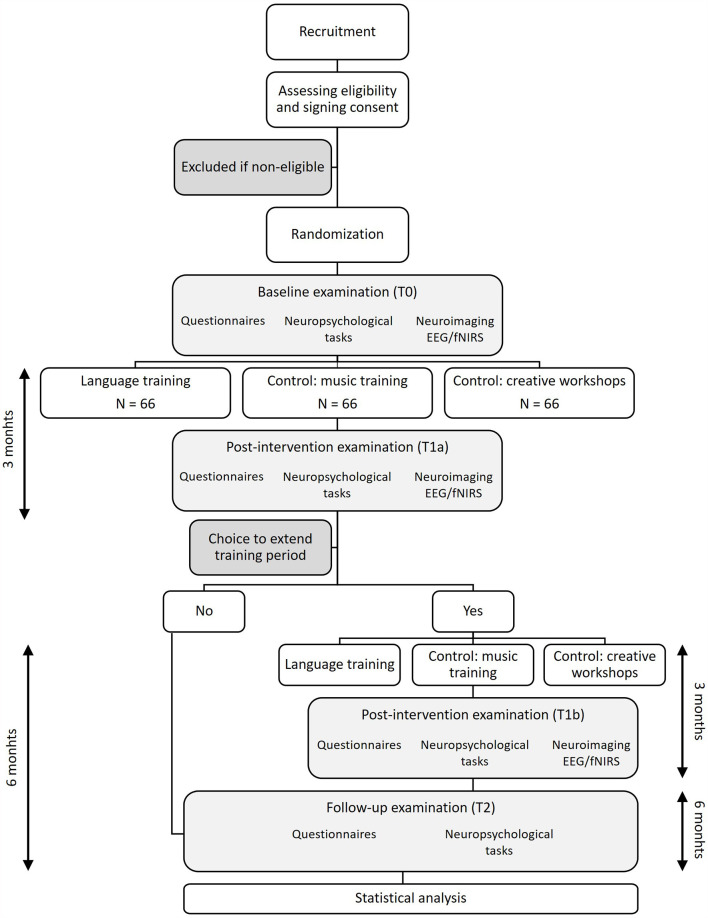
Flow-chart.

The study is designed as an open-label randomized controlled trial with three parallel conditions: A language training intervention, a cognitive control (musical training) intervention and a social control intervention (in the form of creative workshops). The study consists of five phases: (1) a screening phase to determine eligibility to participate; (2) a baseline cognitive, neuropsychological and EEG/fNIRS examination (T0); (3) the first intervention phase (3 months); (4) a post-intervention cognitive, neuropsychological and EEG/fNIRS examination (T1a) 3 months after the start of the intervention. By choice of the participant, the intervention period may be extended to a maximum of 6 months. In this case, phase three is extended and a second post-intervention examination is repeated at the end of this second training period (T1b). The fifth phase (5) is a follow-up examination (T2), which takes place 6 months after the end of the intervention, irrespective of the duration of the intervention. Figure 1 presents an outline of the study design.

### Participants

The study is conducted with elderly at risk for cognitive decline and late-life depression. Based on previous research, this is assumed to be the case in elderly who experience subjective cognitive decline (SCD). SCD is defined by the presence of self-perceived decline in any cognitive domain over the past 5 years (Jessen et al., 2014). Even with normal performance on cognitive tests, SCD has been associated with increased risk for future clinically-assessed cognitive decline (Dufouil et al., 2005; Reisberg et al., 2010; Jessen et al., 2010; Mitchell et al., 2014) and depression (Edmonds et al., 2014; Yates et al., 2017). A total of 198 participants with SCD aged between 65 years and 85 years old are included in the study. Participants are recruited using several strategies: primary (general practitioners) and specialized health care institutions (memory clinics) in the northern part of the Netherlands, organizations for senior citizens, participant platforms such as hersenonderzoek.nl, advertising in regional newspapers and (social) media outreach. Recruitment started in November 2018 and is expected to end in December 2020. To ensure minimal influence of expectation biases, the promotional material does not suggest that there are higher expectations about one intervention compared to another.

#### In-/Exclusion Criteria

A number of inclusion criteria in recruiting subjects are set. Eligible participants should (be):

1. Aged between 65 years and 85 years;
2. Report a presence of subjective cognitive decline: Score > 5 on *Subjective Cognitive Decline Questionnaire* (SCD-q; Rami et al., 2014);
3. Native Dutch speakers;
4. Functionally monolingual (participants do not use any other language other than Dutch in their daily lives and may not be fluent speakers of any other language than Dutch);
5. Not have more than basic English skills (max A2 level on the CEFR scale—Council of Europe, 2001);
6. Not have played a musical instrument over the past 20 years (with the upper acceptable boundary being once a week on average over the past 20 years);
7. Have access to and basic experience with using a computer or tablet given the nature of the intervention (see below);
8. Have an IQ > 85 as estimated using the *Dutch Adult Reading Test* (DART; Schmand et al., 1991).

Exclusion criteria, by contrast, prevent any persons meeting the following conditions to participate in the trial:

1. Presence of objective cognitive decline: Score < 23 on the *Montreal Cognitive Assessment* (MoCA; Nasreddine et al., 2005);
2. Presence of a major DSM-IV disorder or presence thereof in the past 10 years as measured using the Structured Clinical Interview for DSM-IV-R (*SCID-I*, Spitzer et al., 1992);
3. Presence of diagnosed neurological problems (Mild Cognitive Impairment (MCI), dementia, epilepsy, etc.);
4. Daily use of benzodiazepines or antidepressants;
5. Presence of self-reported vision and/or hearing problems that cannot be corrected by vision/hearing aids.

English proficiency skills are assessed in different ways. First, participants are asked to rate their proficiency on a scale of 1–10. Second, based on the descriptions for each skill level (reading, writing, speaking, listening) formulated on the basis of the CEFR can-do scale format, statements are extracted describing something (an activity or conversational act) that a speaker can confidently perform in English. The subject has to indicate whether he/she agrees with these statements on a Likert scale ranging from 1 (strongly disagree) to 5 (strongly agree). If the level of English proficiency remains unclear based on these can-do scales, an online test taken from Cambridge Assessment of English^1^ is sent to the participant.

### Sample Size

Sample size is computed using the program G*Power (Faul et al., 2009). The effect size of the current intervention is unknown, as related studies that have been conducted prior to the current one are of an explorative nature and comprise few participants. A common convention in this case is to set the effect size at a medium threshold, Cohen’s *f* = 0.25 (Cohen, 1969). A lower effect size would not be considered relevant. In order to find a medium effect size on the basis of our outcome measures, with an alpha of 0.05 and a power of 0.80, based on a one-way ANOVA with three groups, a minimum total sample size of 159 participants is required. Considering and allowing for a drop-out of 20%, a total of 198 participants are included in our trial. Past work targeting the effects of cognitive training programs—albeit not language—included a comparable sample size (e.g., Shatil et al., 2014; van de Ven et al., 2015; Buitenweg et al., 2017).

### Randomization

After determining eligibility to participate in the study, participants are assigned to an intervention using a flexible block-wise randomization scheme. Within each block, which differs in size depending on the number of included participants at the moment of randomization, participants are randomly allocated to one of the three study interventions, where every condition occurs equally often. Randomization is done using a computer-generated randomization list in Microsoft Excel. The participants, the researchers and the instructors of the interventions are not blind to treatment condition following allocation. To ensure minimal influence of biases, the information about the current study’s expectations is kept to a minimum for participants and for researchers (student assistants) involved in the testing sessions. Once randomized, participants cannot switch conditions upon discontent with their allocated condition.

### Details of the Interventions

Three intervention arms are included in this study: a language intervention, a music intervention and a social (art) intervention. The language intervention serves as the experimental intervention of interest, while the music and social intervention serve as a high-level active and low-level active control intervention, respectively. All interventions last at least 3 months. After the end of the first training phase, participants can choose to extend the training period to a maximum of 6 months (but depending on availability of instructors, sufficient number of participants who want to continue, etc.). Designing the study in such a way is expected to increase *a priori* motivation because participants do not have to commit to a 6-month training and lowers the threshold to participate but also allows to test the effect of different training durations. In all interventions, participants are asked about their self-rated skills and their motivation relevant to the assigned intervention prior to the start of the intervention and during every class meeting, in order to chart the differences in proficiency and motivation levels over time and relate these to the cognitive and social outcomes of all interventions. Besides that, in all intervention groups, participants log their out-of-class exposure to the trained techniques using a diary during the time they participate in the intervention.

#### Experimental Language Intervention

The cognitive and emotional health effects of a bilingual experience in healthy older adults is investigated through a foreign language training program. During the language intervention, participants are taught basic to intermediate English language skills, with the aim of becoming an independent language user with B1 level on the Common European Framework of Reference for Languages (CEFR). English is chosen as language to be learned based on pilot work carried out within our research group (the Bilingualism and Aging Lab at the University of Groningen: www.balab.nl). This work showed that most elderly would be most motivated to learn English over languages such as Spanish or Italian. Participants with a starting level of A1 or A2 are included in the study, meaning that there can be differences in starting level of individuals. The most important effect to ensue from the language training is that participants start using two languages instead of merely one; they move from being functionally monolingual (albeit where English may be frequently encountered in the landscape) to juggling two languages. This change in language experience and subsequently in cognitive effort needed for resolve conflict is expected to influence cognition. Past work has found such effects to set in very soon after the onset of a foreign language course (see “Introduction” section).

The training is set-up as a blended learning program: participants spend at least 45 min per day, 5 days per week, on (online) learning activities and come in to the University of Groningen for 90-min class meetings with an English language instructor every fortnight during a period of at least 3 months. At home, participants use the provided teaching materials and an online environment to learn and practise the English language. Participants are requested to hand in homework assignments and are given feedback by the instructor. Learning takes place on an individual basis in the participants’ own time and at their own pace. This means that some participants will work through the language materials faster than others. Combined with the fact that participants will have different starting levels, they will likely also end up with different proficiency levels. Due to the large individual differences, class meetings are unrelated to the (online) teaching materials. The class meetings take place in a group setting, with an instructor and several teaching assistants and are mainly aimed at practising communicating with the classmates and speaking the English language. This is achieved by doing group exercises and giving short presentations. During classes, participants are encouraged to talk to each other and to use English only. The small group design ensures that participants can be teamed up with fellow students of the same level in classwork. When possible, a chat group (either in WhatsApp or equivalent platform, or via e-mail) is set up so that participants can chat and ask questions to each other and to the teacher. Just as in class, participants are again encouraged to only speak English in the chat.

The period of 3 months of language training has been suggested as a minimum requirement for cognitive and socio-affective effects to be manifested (Li et al., 2014). The teaching materials and online learning environment are provided by the LOI (*LeidseOnderwijsinstellingen, LOI*). The LOI is a Dutch commercial educational institution that provides training and education at all levels and in difference disciplines. For the purpose of this study, a special online campus is created within the LOI platform. An instruction manual is designed particularly for this online learning environment taking into account the needs of the older adults.

To further tailor the language training towards the specific needs of older adults (see Ramírez Gómez, 2016 for details), the training is embedded within a general communicative language teaching (CLT) model (Richards, 2006) and within that follows a largely implicit teaching method. As such, the training is: (a) focused on speaking and listening; (b) rich in authentic input, e.g., podcasts, movie fragments; (c) focused on chunk learning including idiomatic expressions to ensure immediate relevance; and (d) low in explicit grammar instruction.

#### Control Interventions

A music intervention (high active control) and a no training but social (art) intervention (low active control) are included to respectively assess the unique role of foreign language intervention vs. other complex cognitive intervention programs or social engagement aspects involved in training for seniors.

##### Music Intervention

For optimal comparison, it is important that the music intervention is as similar as possible to the language intervention in format and set-up. Over a period of at least 3 months, participants thus spend a minimum of 45 min per day, 5 days per week, on online learning activities at home and meet with a music instructor for 90-min class meetings every fortnight in learning how to play the guitar. In other words, a blended learning program is implemented which mirrors the intensity of the language training. Also in line with the language condition, teaching materials are provided by the LOI. Online assignments also need to be handed in via the LOI platform and are marked by the music instructor. Although the LOI also provides similar music courses for different musical instruments, a guitar course is the most practical one: a guitar can be provided to the participants as part of the study, which would not have been possible for the other instruments for financial and practical reasons.

##### Social Intervention

In the social intervention, participants meet every other week for 90-min over a period of at least 3 months, similar to the other two interventions. During these meetings, different creative workshops are offered, where participants engage in craft activities led by instructors. Examples include painting, woodcrafts, etc. In the social control group, participants are not expected to continue their activities at home and no online training is provided, contrary to the language and music conditions. Out-of-class activity is monitored by means of a diary, similar to the other two conditions. This low-level active control condition involves the same level of social interaction as the other two training interventions, but the amount of complex skill learning is kept to a minimum.

### Materials

An overview of all interviews, questionnaires and neuropsychological assessments is provided in Table 1.

**Table 1:** Measurement items and points of data capture.

Instrument	Screening	Baseline T0	Post-intervention T1a	Post-intervention T1b	Follow-up T2
		0 months	3 months	6 months	9/12 months
Demographics	x
Motivation	x
Language, music and creative skills	x		x	x	x
SCID-I	x				x
SCD-q	x				
MoCA	x	x	x	x	x
GDS	x	x	x	x	x
LARSS		x	x	x	x
ERQ		x	x	x	x
AES		x	x	x	x
CFQ		x	x	x	x
TIPI		x	x	x	x
WHOQOL-BREF		x	x	x	x
Loneliness scale		x	x	x	x
Course evaluation			x	x	
CRIq		x			x
TMT		x	x	x	x
DSST (WAIS-IV)		x	x	x	x
Digit span tests (WAIS-IV)		x	x	x	x
Verbal fluency test L1		x	x	x	x
mWCST		x	x	x	x
Color-shape switch task		x	x	x	x
					
EEG/fNIRS		x	x	x	
**Only for participants in the language intervention**
Verbal fluency test L2		x	x	x	x
PPVT		x	x	x	x
IELTS		x	x	x	x

#### Primary Outcomes

The main research question of this study is whether a language training can uniquely enhance cognitive flexibility in elderly with SCD. The primary outcome is therefore the change in cognitive flexibility following the interventions and differences herein. Tasks used in the current study to measure cognitive flexibility on a behavioral level include the *Trail Making Test* (TMT), the digit span tests and symbol substitution of the Wechsler Adult Intelligence Scale (WAIS-IV; Wechsler, 2008), the *modified Wisconsin Card Sorting Test (mWCST)* and a *color-shape switching task*. The color-shape switching task is designed and controlled in OpenSesame version 3.1.9 (Mathôt et al., 2012). Four different versions of the color-shape switching task are used in a counterbalanced manner, which differ in the order of task blocks and the hand participants use to respond to the different blocks.

To complement the behavioral results on cognitive flexibility, combined EEG and fNIRS methods are used in an explorative nature to assess cognitive flexibility on a neuronal level during the color-shape switching task. These methods provide information on whether the increase in cognitive flexibility, as a result of the language training, also results in changes in neuronal networks in the frontal areas. EEG and fNIRS are easy to implement and less invasive than other imaging methods such as fMRI and are therefore less burdensome for elderly. Specific EEG outcomes are changes in power coherence of frequency bands, especially the theta-band as an indicator of processes involved in cognitive flexibility. NIRS outcomes are changes in the blood oxygenation level dependent (BOLD) response in the dlPFC and vlPFC.

#### Secondary Outcomes

Secondary study parameters include changes in (mental) health outcomes, focusing on subjective and objective cognitive changes, depressive symptoms, emotion regulation, rumination, apathy, well-being and loneliness following the language intervention. Subjective cognitive functioning is quantified using the *Cognitive Failures Questionnaire* (CFQ; Broadbent et al., 1982). Objective cognitive functioning is assessed using the *Montreal Cognitive Assessment* (MoCA; Nasreddine et al., 2005). The *Geriatric Depression Scale* (GDS; Yesavage et al., 1983) is used to measure the severity of current depressive symptoms and the course of such symptoms during the study is monitored using the Structured Clinical Interview for DSM-IV disorders (SCID-I; Spitzer et al., 1992). The *Emotion Regulation Questionnaire* (ERQ; Gross and John, 2003) measures the use of two emotion regulation strategies: suppression and reappraisal and the *Leuven Adaptation of the Rumination on Sadness Scale* (LARSS; Raes et al., 2007) is employed to measure ruminative thinking of sadness. Furthermore, apathy is assessed using the *Apathy Evaluation Scale* (AES; Marin et al., 1991), quality of life is operationalized using the abbreviated version of the *World Health Organization Quality of Life questionnaire* (WHOQOL-bref; The WHOQOL Group, 1998), and loneliness using the *Loneliness scale* (De Jong-Gierveld and Van Tilburg, 2006). For the MoCA, three different version are used, and these are counterbalanced between subjects.

#### Other Outcomes

Several other, potentially confounding, variables are measured, such as cognitive reserve, *a priori* motivation to learn (learning in general and learning English/guitar/creative skills in particular), personality traits, digital proficiency, self-reported skills in additional languages or dialects other than the mother tongue, and music and creativity, and questions regarding evaluation of the course (e.g., group cohesion, teacher evaluation). Cognitive reserve is measured on the basis of the *Cognitive Reserve Index Questionnaire* (CRIq; Nucci et al., 2012), which elicits information about participants’ adult life. The above-mentioned measures serve to provide additional information to interpret and understand the effects induced by the language intervention vis-à-vis the other interventions and to understand the changes in neurocognitive functioning. Also, language proficiency measures are obtained for participants randomized to the language intervention to monitor their progress. Although not a primary outcome variable, proficiency progress may influence the cognitive flexibility and mental health outcomes to ensue from this study. Language proficiency in the learned language English (L2) is tested using the *Peabody Picture Vocabulary Test* (PPVT; Dunn et al., 2005), which assesses receptive English vocabulary. Productive vocabulary in the L1 Dutch and L2 English is measured using a phonetic verbal fluency task, where as many words starting with a given letter have to be named in the timespan of 1 min for three different letters. A different combination of letters is used for each examination, ensuring that the different versions are counterbalanced and that each subject performs each version at least once. The English version of the task uses a combination of the letters CFL, PWR and FAS. For the Dutch version, the letter combinations DAT, KOM and PGR are used. Proficiency in speaking and listening in English is further assessed using methods based on the *International English Language Testing System* (IELTS).

#### Equipment

EEG is recorded using 34 Ag|AgCl electrodes mounted on a textile cap (EasyCap, Herrsching, Germany). The electrodes are arranged in equidistant positions according to the international 10/20 system. EEG recordings are performed using an ANT Neuro amplifier system (Advanced Neuro Technologies B.V., Enschede, The Netherlands) at a sampling frequency of 512 Hz. Electrode impedances are kept under 7 kΩ. Recordings are made with AFz and FCz as ground and reference electrodes, respectively. Two extra electrodes are placed at both mastoids and are used to re-reference the EEG signal offline. Additionally, electrodes are placed above and below the left eye and laterally at the sides of both eyes to measure horizontal and vertical eye movements.

Continuous optical signals are recorded using the NIRScout 1624 imaging system and NIRStar software (NIRx, Berlin, Germany) equipment. The signal is collected at a sampling rate of approximately 7.8 Hz. In total, 16 optodes (eight sources, eight detectors) with dual-wavelength (760 nm and 850 nm) are symmetrically placed on the head, to measure both hemispheres. The optodes are placed in pre-set locations in the same cap used for the EEG measurements, using landmarks of the 10-20 EEG electrode placement system, around and among the locations FP1/2, F3/4, F7/8, FC3/4, FT7/8. These locations cover the dlPFC and vlPFC. As a result, data from 18 channels (nine for each hemisphere) are recorded.

### Procedure

All data are collected at the University Medical Center Groningen, the Netherlands. Individuals who express an interest in participating and seem to fulfil the criteria are invited for a screening. During the screening, and when the study is clear to the participants, the informed consent forms are signed and screening materials, including assessment of motivation for the three interventions before intervention assignment (see Table 1) are administered. Following this initial screening, participants fulfilling eligibility criteria (see criteria above) are randomized in a block-wise fashion to one of the three intervention groups. It is expected that six or seven blocks of randomization are required to reach the total target sample size of 198 participants. Randomization takes place when a sufficient number of participants are included to form a group of at least six to at most 15 participants for each intervention. This group size is chosen to ensure social interaction and smaller group activities as well as personal attention (which is harder when the groups become too large).

The baseline testing takes place within 1 week prior or following the start of the intervention. Research assistants’ expectations of the superiority of any of the interventions have been kept to a minimum. Preceding the testing session, most of the questionnaires are sent to the home address of the participant to reduce burden on the day of testing. During the baseline assessment, the GDS, MoCA, CRIq and the cognitive tests (WAIS-IV subtests, verbal fluency L1, TMT and mWCST) are administered. Finally, EEG/fNIRS measurements are recorded during the color-shape switching task and an eyes-open resting state session. All of this is done in a dimly lit and shielded room. The language proficiency tests (PPVT, verbal fluency L2 and IELTS) are only administered for the participants randomized to the language training and are conducted on a different day, in a separate testing session and by the language course instructor.

For a period of at least 3 months, participants engage in their assigned interventions. The training period may be extended to a maximum of 6 months by choice of the participant. The post-intervention examination (T1a) is scheduled 3 months after the start of the intervention and is completed by all participants. This examination is the same as the baseline examination but excludes the CRIq and contains additional questions about self-perceived proficiency and course evaluation. The post-intervention examination is repeated if the training period is extended (T1b) and is scheduled at the end of the second training period. Finally, a follow-up examination (T2), 6 months after the end of the intervention, sees a repetition of the previous post-intervention examinations plus a shortened version of the SCID-I interview and the CRIq, but excludes the fNIRS/EEG measures and course evaluation questions.

After the final examination has taken place, participants receive a gift card of 25 euros to thank them for their participation in the study. Travel cost reimbursement is also available, if necessary.

### Statistical Analysis

#### Behavioral Data

Descriptive characteristics of the different intervention groups are summarized using simple descriptive statistics and compared among groups to assess balance of covariates (for e.g., age, gender, etc.), using one-way ANOVA for continuous variables and Chi-Square tests for categorical variables. Appropriate nonparametric tests are used in case assumptions are violated. The effectiveness of the different interventions is assessed by examining differences in scores between the different times of assessment for each of the primary outcome variables measuring cognitive flexibility. Multilevel linear mixed effects modeling procedures are used for this aim with time (baseline, post-intervention I/II and follow-up) at Level 1, individual participants at Level 2 and intervention (three levels: language/music/art) as independent variable. Age, sex, educational level and *a priori* motivation are added as covariates in follow-up steps. Besides that, T-tests are used to specifically compare the two high-level interventions (language and music intervention) to the low-level intervention (art) and to compare the language and music intervention to each other. The alpha level is set at 0.05. Standard statistical packages including the Statistical Package for the Social Sciences (SPSS) and R (R Core Team, 2006) are used for analysis.

#### EEG/fNIRS Data

Processing and analysis of the EEG data are performed using the FieldTrip toolbox for EEG/MEG-analysis (Oostenveld et al., 2011). The effect of training on the power and coherence of the EEG-signal during the cognitive flexibility task, with a specific focus on the theta-band, is analyzed within-subjects and compared among groups (language vs. music vs. creative workshops). Appropriate non-parametrical tests are used in case of a non a normal distribution. Processing and analysis of the fNIRS signal, is performed using the NIRS toolbox (or NIRS Brain AnalyzIR toolbox; Santosa et al., 2018). Oxygenated hemoglobin (Oxy-Hb) levels in de dlPFC and vlPFC are analyzed using a mixed effect models analysis, to analyze the effect of the training over time (within-subjects) and to compare among groups (language vs. music vs. creative workshops). A false discovery rate (FDR) correction with a significance level set at 0.05 was used to control for multiple comparisons (Benjamini and Hochberg, 1995).

**Adjustments due to COVID-19 pandemic.**

• creative workshop classes were omitted for the duration of physical distance related restrictions.
• classes for the language and music intervention were organized online via video calls.
• measurements took place via video calling.
• mWCST was initially administered using playing cards. For the duration of the restrictions this task was administered as a computer task.
• fNIRS/EEG measurements were omitted for the duration of physical distance related restrictions.
• influence of COVID-19 adjustments (online/offline) is explored in the analysis of the study.

## Discussion

Current controversies about the bilingual advantage exist due to ambiguous findings from previous studies. If a bilingual experience affects cognitive control, and if so, under which conditions (pertaining, crucially, also to differences in bilingual experiences) is therefore a question that needs to be addressed, perhaps in a different way than by comparing bilingual and monolingual groups, as has been common practice in the past. In this experimental randomized controlled trial, this is achieved by comparing the effectiveness of a short-term but intense foreign language training to other cognitive and/or social stimulating activities in old age, in seniors at risk for cognitive decline and late-life depression. This study is the first to combine a wide range of complementary language proficiency, socio-affective, cognitive and brain measures in a comprehensive design comprising a large number of participants. This multidisciplinary approach allows for the investigation of mechanisms underlying the possible effects of foreign language learning.

If language training for seniors proves effective in improving cognitive flexibility and provoking changes in its neural underpinnings—ultimately promoting cognitive reserve—this can have important clinical implications. Cognitive decline is common among elderly, but there are currently few effective prevention methods available and patients with memory complaints are often left empty-handed. The results of this study could possibly contribute to the development of effective language learning interventions for seniors aimed at reducing the risk for multiple old-age disorders such as cognitive decline and late-life depression. Although out of scope of the current study, other age groups might also benefit from the improvement in cognitive flexibility resulting from learning a new language and it could be applied in rehabilitation in other disorders where cognitive flexibility is compromised, such as in schizophrenia (Waltz, 2017), traumatic brain injury (McDonald et al., 2002) or chemo brain (Yamada et al., 2010). Future studies therefore should focus on the generalizability of the study’s potential effects.

The results of this study might furthermore lead to insights into the uniqueness of language learning, compared to other complex skill learning and social interaction, in enhancing cognitive flexibility and mental health. Many studies suffer from lack of adequate control conditions, making it impossible to disentangle the effect of speaking (or indeed learning) multiple languages from other influences. It should be noted, however, that the language and music intervention both use blended learning programs: a part is online and computer-mediated, and a part takes place in physical classes. There is evidence that these kind of computer-assisted learning interventions themselves can lead to improvements in cognition in elderly (González et al., 2015). To control for this, the amount of online activities is kept constant across both the language and music interventions and logged by means of diaries.

A further strength of this study lies in the intensity of the language intervention completed by a large number of participants. The overall investment per participant is on average 4.5 h per week over the course of 3 months. This is in line with suggestions that for cognitive effects to become overtly noticeable a minimum of 5 h of language training per week (Bak et al., 2016) and a minimum of 3 months training (Li et al., 2014) is necessary. However, as the field of language training studies for elderly is only emerging, little is known about which choices can best be made regarding training intensity in this age cohort. Earlier language training studies have used both less intense and more intense training paradigms; however, this has not led to a clear picture of which training intensity leads to cognitive effects (Pot et al., 2019). The same is true for other important variables such as the language taught and the format of the language course (i.e., teaching method). As long as these questions remain unanswered, it is not clear which choices are most likely to produce the expected results. Although the current study was not set up to evaluate feasibility and acceptability of the language intervention, these features will be monitored and reported. The current study will therefore hopefully be a step in the right direction and provide the field with useful information on some of these parameters. By allowing an investigation of the exposure-response relationship between language learning and its effects through the possibility of extending the training period to 6 months, the current study can, for example, provide insights in the needed intensity for effects to emerge.

At the same time, this study has several limitations that need to be considered. First, participant biases are likely to play a role. In demanding and intensive studies, such as this one, individuals must be motivated to actively participate in the course. Indicating interest in participation can therefore already be a bias because it can say something about motivation and self-initiative. Individuals who are interested in participating are presumably already quite active in their daily lifestyle and activities. There is likely also a bias in the level of education; individuals with a lower level of education are inclined to be selected for participation because higher educated individuals are more likely to be proficient in English or to play a musical instrument and therefore do not meet the inclusion criteria. Second, a methodological difficulty is the drop-out risk that is associated with any longitudinal study with follow-up assessments. This is, however, partially controlled for by including more participants than strictly needed (from a statistical power perspective) in case of a conservatively estimated 20% attrition rate. Third, there are several factors that may complicate participant inclusion. The study takes place in the northern Netherlands, a geographical area rich in dialects and languages. Because of this, it can be difficult to find eligible participants that comply with the inclusion criteria (most notably that of functional monolingualism). Additionally, the blended-learning set-up of the study requires participants to carry out a large portion of their learning at home using an online learning environment. Especially for the elderly, this can be difficult to navigate. Elderly participants might fear using online learning methods, which prevents them from participating, or they might find it too difficult and quit the study which further increases the drop-out risk. However, by offering a specially designed instruction manual this is largely remedied.

In conclusion, the current study is designed to test whether language learning is unique in improving cognitive flexibility and psychological health, thereby promoting healthy aging and advance the understanding of the effect of a bilingual experience. Future longitudinal studies are needed to study the long-term clinical impact and to confirm whether a language training can actually prevent cognitive decline in aging populations.

## Ethics Statement

The studies involving human participants were reviewed and approved by Medical Ethical Committee University Medical Center Groningen. The patients/participants provided their written informed consent to participate in this study.

## Author Contributions

M-JT, MK, and SN were responsible to the design of the study. SN wrote the study protocol and drafted the manuscript. M-JT and MK were involved in critically revising and finalizing the draft. All authors were involved in reviewing and finalizing the draft. All authors contributed to the article and approved the submitted version.

## Conflict of Interest

The authors declare that the research was conducted in the absence of any commercial or financial relationships that could be construed as a potential conflict of interest.

## Footnotes

^1^https://www.cambridgeenglish.org/test-your-english/general-english/

## Abbreviations

AES: Apathy Evaluation Scale
ANOVA: analysis of variance
BOLD: blood oxygenation level dependent
CEFR: Common European Framework of Reference for Languages
CLT: Communicative Language Teaching
CFQ: Cognitive Failures Questionnaire
DART: Dutch Adult Reading Test
dlPFC: Dorsolateral Prefrontal Cortex
EEG: Electroencephalography
ERQ: Emotion Regulation Questionnaire
fNIRS: functional Near Infrared Spectroscopy
GDS: Geriatric Depression Scale
IFG: Inferior Frontal Gyrus
L1: Native language
L2: Second (foreign) language
LARSS: Leuven Adaptation of the Rumination on Sadness Scale
MCI: Mild Cognitive Impairment
MFG: Medial Frontal Gyrus
MoCA: Montreal Cognitive Assessment
mWCST: modified Wisconsin Card Sorting Test
NTR: Dutch Clinical Trial Register
Oxy-Hb: Oxygenated hemoglobin
PPVT: Peabody Picture Vocabulary Test
SCD: Subjective Cognitive Decline
SCDq: Subjective Cognitive Decline questionnaire
SCID-I: Structured Clinical Interview for DSM-IV Axis I disorders
SFG: Superior Frontal Gyrus
T0: timepoint 0, baseline
T1a: timepoint 1b, post-intervention measurement 3 months after T0
T1b: timepoint 1b, optionally, post-intervention measurement maximal 6 months after T0
T2: timepoint 2, 6-month follow-up post-intervention
TMT: Trial Making Test
UMCG: University Medical Center Groningen
vlPFC: Ventrolateral Prefrontal Cortex
WAIS-IV: Wechsler Adult Intelligence Scale-IV
WHOQOL-bref: World Health Organization Quality of Life questionnaire abbreviated.
